# Steatosis, Steatohepatitis and Cancer Immunotherapy: An Intricate Story

**DOI:** 10.3390/ijms222312947

**Published:** 2021-11-30

**Authors:** Mauro Cataldi, Federica Manco, Giovanni Tarantino

**Affiliations:** 1 Department of Neuroscience, Reproductive Medicine and Dentistry, Section of Pharmacology, Federico II University, Medical School of Naples, 80131 Naples, Italy; cataldi@unina.it (M.C.); fede.manco3@hotmail.com (F.M.); 2 Department of Clinical Medicine and Surgery, Federico II University, Medical School of Naples, 80131 Naples, Italy

**Keywords:** steatosis, steatohepatitis, hepatocellular carcinoma, immune checkpoint inhibitors

## Abstract

Immune checkpoint inhibitors represent one of the most significant recent advances in clinical oncology, since they dramatically improved the prognosis of deadly cancers such as melanomas and lung cancer. Treatment with these drugs may be complicated by the occurrence of clinically-relevant adverse drug reactions, most of which are immune-mediated, such as pneumonitis, colitis, endocrinopathies, nephritis, Stevens Johnson syndrome and toxic epidermal necrolysis. Drug-induced steatosis and steatohepatitis are not included among the typical forms of cancer immunotherapy-induced liver toxicity, which, instead, usually occurs as a panlobular hepatitis with prominent lymphocytic infiltrates. Nonetheless, non-alcoholic fatty liver disease is a risk factor for immunotherapy-induced hepatitis, and steatosis and steatohepatitis are frequently observed in this condition. In the present review we discuss how these pathology findings could be explained in the context of current models suggesting immune-mediated pathogenesis for steatohepatitis. We also review evidence suggesting that in patients with hepatocellular carcinoma, the presence of steatosis or steatohepatitis could predict a poor therapeutic response to these agents. How these findings could fit with immune-mediated mechanisms of these liver diseases will also be discussed.

## 1. Introduction

Cancer cells evade host immune responses by activating specific immune tolerance mechanisms, which include key proteins of the immune checkpoints physiologically involved in self-tolerance. These mechanisms consist of corepressor proteins on antigen presenting cells and their ligand receptors in T-lymphocytes, whose engagement reduces T-cell activation and modulate immune responses. By impairing these tolerance systems, anticancer agents of a new class, immune checkpoint inhibitors (ICIs), restore the immune response against tumors and induce clinical responses which are often impressive [1,2]. Of the many corepressor systems that have been identified so far, only two have been targeted with ICIs, CTLA-4 and PD1/PD-L1. CTL-4 is expressed on T-lymphocytes and acts as a decoy receptor competing with CD28, a lymphocyte coactivator receptor, for the binding to CD80 or CD86—its ligands expressed by antigen presenting cells—and therefore, it prevents T-lymphocytes’ activation. PD1 are corepressor receptors expressed on lymphocytes whose activation by PD-L1 and PD-L2 proteins (on/in cancer cells or on antigen presenting cells, respectively) reduces T-cell activity by triggering a tyrosine phosphatase signaling cascade [1,2]. It is noteworthy that most of the knowledge on these the mechanisms of action has come from in vitro studies and is therefore still hypothetical.

ICIs have revolutionized cancer therapy since their licensing, by dramatically improving the prognosis of patients with responsive tumors. Unfortunately, due to their property of reducing self-tolerance, they may also induce severe systemic immune-mediated toxicities whose spectrum is large and which cause many organ-specific immune diseases, such as thyroiditis, nephritis, hypophisitis, colitis and more rarely, a devastating systemic autoimmune syndrome which resembles graft versus host disease [3,4]. Hepatotoxicity is common in patients treated with ICIs, and its prevalence is reported to be around 2% for monotherapy with anti PD-1 anti PD-L1 antibodies and up to 30% with combined therapy against PD-1/PDL-1 and CTL-A4 (Table 1) [5,6,7,8,9]. The meta-analysis by Wang et al. (2017) [10] showed that an increase in AST and/or in ALT concentration occurred in 2–5% of patients receiving nivolumab, pembrolizumab and atezolizumab, whereas less than 2% of them showed clinically evident hepatitis, which was severe in less than 1% of them. The systematic review by Peeraphatdit et al. (2020) [11] reported a prevalence of hepatotoxicity ranging from 0.7% to 16% of patients depending on which ICI was used (0.7–2.1% with anti- PD-1 antibodies; 0.9–12% with anti-PD-L1 and standard-dose anti-CTLA-4 antibodies; and 13% and 16%, with combined anti CTLA-4/PD-1 and high-dose anti CTLA-4 therapies, respectively). Even though the usual presentation of ICI-induced hepatotoxicity is an immune-mediated hepatitis with hepatocellular damage and immune cell infiltration, sometimes the pathological features of cholangitis or a mixed pattern hepatitis with cholangitis are observed [11,12,13].

Drug-induced liver injury (DILI) is a potential serious adverse reaction of many drugs. DILI caused by xenobiotics is profoundly different from that induced by antibodies, since the first (such as thioacetamide or carbon tetrachloride) mostly act by causing direct damage to hepatocytes with secondary involvement of liver macrophages [58], whereas the second directly interact with dendrite cells and liver macrophages [59], as we will discuss in deep later. DILI can present in the form of non-alcoholic fatty liver disease (NAFLD) [60], which, in turn, increases the susceptibility to drug-induced hepatotoxicity, and therefore, is a risk factor for DILI [61]. Different classifications of NAFLD-inducing drugs have been proposed, but none of them is universally accepted. Grieco et al. (2015) [62] suggested a classification into three groups which has the advantage of giving emphasis to the clinical consequences of the liver damage that they induce: 1. drugs that induce metabolic changes and can precipitate latent NASH, which needs additional triggering factors to become clinically evident (e.g., tamoxifen); 2. drugs that cause steatosis and steatohepatitis independently from any concomitant triggering factor (e.g., amiodarone and perhexiline maleate); and 3. drugs that induce sporadic events of steatosis/steatohepatitis (e.g., carbamazepine). It is still unclear whether ICIs should be included among NAFLD-inducing drugs, and if yes, in which of the above-mentioned categories they fit best. The term NAFLD was introduced in the 80’s by Schaffner and coll. [63] to describe a clinical condition characterized by histopathological alterations which are similar to those observed in alcoholic liver disease but occur in the absence of alcohol abuse. These alterations include fat accumulation in the hepatocytes (steatosis, NAFL), which may progress to steatohepatitis (NASH), which is characterized by significant liver inflammation associated with macrovesicular steatosis and hepatocellular ballooning. NASH further evolves to cirrhosis and liver fibrosis in 10–20% of patients [64]. However, it also completely resolves in 10.9% of cases, and regresses to borderline steatohepatitis—a condition showing only some of the pathological characteristics of NASH- or NAFL in 20.3% and 11.2% of patients, respectively [65]. Drugs are responsible for only about 2% of NAFLD cases [66,67], since this condition is generally associated with metabolic disorders. To better emphasize the metabolic pathogenesis of this disease, two position papers from experts of the field suggested in 2020 that the term NAFLD should be replaced with MAFLD (metabolic associated fatty liver disease) [68,69,70]. However, in the present review, we use the old terminology of NASH and NAFLD, since although many drugs may induce metabolic disturbances which could have be involved in causing hepatotoxicity, it is unclear whether and when the term MAFLD could be used for “drug-induced NAFLD.” Moreover, skepticism has been raised on the real benefits of replacing the universally known acronym NAFLD with a term, MAFLD, which could not adequately fit with the evidence that multiple factors besides metabolic disturbances could cooperate in the onset/progression of this disease [71,72].

## 2. Aim of the Review

We reviewed evidence on the possible connection between NASH and ICIs. We show that not only may NAFLD and NASH occur in the context of ICI hepatotoxicity, but also that preexisting NAFLD influences the susceptibility to ICI-induced hepatotoxicity and the efficacy of these drugs in the treatment of hepatocellular carcinoma (HCC).

## 3. Methods

To prepare this narrative review, we interrogated PubMed (https://pubmed.ncbi.nlm.nih.gov/ (accessed on 10 October 2021)), Scopus (https://www.scopus.com/search/form.uri?display=basic&zone=header&origin=#basic (accessed on 10 October 2021)) and Embase (https://www.embase.com/ (accessed on 10 October 2021)) to track recent evidence using the following keywords: immune checkpoint inhibitors, NASH, NAFLD, hepatic steatosis, steatohepatitis, hepatocellular carcinoma, anti-VEGF drugs.

## 4. Liver Biopsy Shows That Intrahepatic Fat Accumulation Is Common in Patients Treated with ICIs

Cohen et al. (2021) [13] described the pathology findings in 60 patients who underwent liver biopsies because they showed elevated circulating liver enzymes during therapy with ICIs. Three main patterns were identified: 1. a predominantly hepatitic pattern of injury, with lobular inflammation (mainly centrilobular) and infiltration of histiocytes and lymphocytes—sometimes with granuloma formation; 2. a predominantly cholangitic pattern with minimal or no lobular inflammation; and 3. a mixed hepatocellular and cholangitic pattern of injury in which the histological features of the two patterns previously described do coexist. Fatty infiltration was observed in about 40% of patients with the predominantly hepatitic pattern, and importantly, in 60% of them it was limited to the areas where hepatitis was detected, suggesting that it was related to the ongoing lobular inflammation. In a small subset of three patients (5%) a steatohepatitic pattern indistinguishable from NAFLD was observed.

Similar results have been reported on a smaller series of eight patients by Zhang et al. (2020) [36]. They observed that lobular hepatitis was the most prevalent pathology, finding it in six patients out of eight, and that in 50% of cases it was accompanied by macrovescicular steatosis. In addition, in one of the two patients not showing lobular hepatitis, the histological examination showed a pure steatohepatitis pattern. This patient was obese, and he had undergone a previous liver biopsy before starting therapy with nivolumab. Compared with the biopsy performed during immunotherapy, this pretreatment biopsy only showed mild macrovescicular steatosis with no statohepatitis. According to the authors, this finding could suggest that in this patient, steatohepatitis was a complication of immunotherapy and not a preexisting “background” condition due to obesity.

## 5. NAFLD as a Potential Risk Factor for ICI-Induced Hepatotoxicity

A recent clinical investigation by Sawada et al. (2020) [73] reported evidence suggesting that NAFLD could represent a risk factor for ICI-induced hepatotoxicity. The authors retrospectively looked for ICI-induced predisposing factors in the medical records of 135 patients who received the anti PD-1 antibodies nivolumab or pembrolizumab at a single institution in Japan for the treatment of various types of solid tumors, including non-small-cell lung cancer, malignant melanoma, gastric cancer, renal cell carcinoma, urothelial carcinoma, head and neck squamous cell carcinoma and malignant pleural mesothelioma. Grade 2 or higher hepatotoxicity according to the Common Toxicity Criteria for Adverse Events of the National Cancer Institute occurred in 36 of these patients. Among the many variables that were included in univariate and multivariate Cox hazard analysis (age, gender, BMI > 22.3, serum albumin, baseline liver enzyme level and the presence of liver metastases), the only one that was significantly associated with ICI-induced hepatotoxicity was the presence of NAFLD, which was associated with a hazard ratio of 29.34.

Recently, Hamid et al. [74] reported in an abstract to confirmatory data on the association between NAFLD and ICI-induced hepatotoxicity from a large series of 18,150 patients. They found that NAFLD significantly increased the odds ratio of undergoing hepatotoxicity from 2.34 to 3.62.

The reasons for the association between NAFLD and ICI-induced hepatotoxicity are unclear. A possible explanation is that because of some NAFLD-related metabolic dysfunction of hepatocytes, either more free radicals are generated in these cells or toxic environmental substances are less efficiently inactivated. This process would lead to the enhanced production of neoantigens and ultimately to immune aggression to the liver, especially if self-tolerance is impaired by ICIs. An alternative explanation is that ICIs could activate or enhance immune mechanisms that are normally involved in the pathogenesis of NAFLD. This hypothesis is explored in the next section.

## 6. Similarities between the Pathogenetic Mechanisms of NASH and Those of ICI-Induced Hepatotoxicity

A wealth of experimental evidence suggests that both innate and adaptive immunity and the inflammatory responses that they cause have crucial roles in the progression of steatosis to steatohepatitis and its complications [75,76]. According to the classic “two-hit” hypothesis of NASH pathogenesis, two hits are required for the occurrence of NASH. Tissue inflammation with cytokine release is the second one, the first being lipid accumulation in liver cells [77].

Intracellular lipid accumulation is supposed to cause a specific form of liver cell damage, lipotoxicity, which involves ensuing mitochondrial dysfunction, and consequently, the release of radical oxygen species (ROS) and lipid peroxidation products. It is important, however, to underline that fat storage in the hepatocytes may not be sufficient, per se, to cause hepatocyte damage unless additional noxae such as alcoholic beverages or hepatotoxic drugs potentiate its effects [78]. Radical oxygen species (ROS) and lipid peroxidation products, together with damage-associated molecular patterns (DAMPS), which are released by damaged hepatocytes, are supposed to trigger innate immunity, and consequently, tissue inflammation (the second hit of the “two hit hypothesis”). More recently, evidence has been reported that tissue inflammation could precede lipid accumulation and cause it in a sort of “reversed” two-hit manner [79,80]. Still other authors have formulated the “multiple hit” hypothesis. It considers multiple insults acting together on genetically predisposed subjects, providing a more accurate explanation of NAFLD pathogenesis [81].

Whatever the real chain of events, the final result is the activation of resident innate immunity cells—including macrophages and Kupfer cells—and the recruitment to the liver of circulating neutrophils, monocytes, natural killer (NK) and natural killer T (NKT) cells. In parallel, proinflammatory cytokines, including TNFα,IL-1β and IL-6, and chemokines such as IL-12, CC-chemokine ligand 2 (CCL2) and CXC-chemokine ligand 9 (CXCL9), are released and contribute to amplifying innate immunity and recruit monocytes and T and B lymphocytes, the cell types which mediate adaptive responses once that they get activated [82]. Interestingly, TNF-α is physiologically released in response to systemic inflammation as a positive acute-phase reactant; this main cytokine becomes toxic for the liver only in the presence of additional toxic factors, such as cycloheximide [83]. Dendritic cells have a key role in bridging innate and adaptive immunity in NASH. In normal conditions, these cells present gut-derived antigens to T lymphocytes in a tolerogenic way. By contrast, when lipotoxicity establishes an inflammatory environment in the liver, these cells switch from the tolerogenic immature phenotype to an activated phenotype, in which form they not only further promote liver inflammation but also trigger adaptive T-cell mediated responses. Specifically, adaptive immune response is directed either toward gut-derived antigens and neoantigens generated by free radical reaction with endogenous compounds (oxidative stress-derived epitopes, OSE). Remarkably, activated dendritic cells also accumulate lipids intracellularly and may, therefore, further promote lipotoxicity [84,85]. Adaptive immune responses are mediated by dendritic cell-mediated activation of CD4+ and CD8+ effector T lymphocytes which is finely modulated by the concomitant activation of regulatory T-cells (T_reg_). The extent of liver damage progression will be the result of the balance between the number/activity of T_reg_ and effector T-cells. More specifically, in recent years CD4+ T helper type 17 (Th17) cells that release IL-17, a crucial proinflammatory cytokine responsible for NAS progression [86], emerged as the primary lymphocyte subtype responsible for liver inflammation, and the ratio between Th17 and Treg as the major determinant for the progression of NASH [87].

The relevance of the adaptive immune responses in the progression of NASH is supported by the remarkable benefits of immunosuppressive therapies in this disease. Interestingly, gut restricted immunosuppression—for instance, with anti-CD3 antibodies given orally—appears more promising than systemic immunosuppression because of its higher tolerability [88]. Acting in the gut, these antibodies promote the induction of a specific subset of T_regs_, CD4+/CD25-latency associated peptide (LAP)+ T-cells, which migrate to lymph nodes where they exert their immunosuppressive effects, finally leading to a decrease in the Th1-Th17/T_reg_ ratio and to an improvement of NASH [89].

The adaptive immune mechanisms involved in the pathogenesis of NASH and in its complications establish a critical link between these diseases and the hepatotoxicity caused by ICIs. Indeed, these drugs may interfere with the activation of adaptive immunity in the liver at multiple levels. Anti-CTLA-4 monoclonal antibodies may disturb the activity of dendritic cells. Indeed, dendritic cells express the CTLA-4 receptors CD80/CD86, which are induced upon their switching to an activated phenotype and are involved in the activity of these cells in presenting antigens to T lymphocytes [85,90]. In addition, ICIs may alter Th1:Th17 and T_reg_ cell number/activity [91]. It is well known that by suppressing CTLA-4 or PD1/PDL1-mediated corepressor signals, ICIs enhance T effector cell activity, and this represents the main mechanism behind their anticancer activity [1,2]. Moreover, ICIs may also reduce the activity of Tregs or decrease their number. More specifically, the anti-CTLA-4 antibodies ipilimumab and tremelimumab may deplete T_regs_ by triggering Fc-dependent cytotoxicity upon interaction with CTLA-4, which is constitutively expressed on these cells [91,92,93,94]. Interestingly, CTLA-4 has also an important functional role in T_regs_ cells, as demonstrated by their dysfunction in CTLA-4 knockout mice [95], and therefore, anti CTL-4 antibodies may impair T_regs_ activity by CTLA-4 immunoneutralization. Whether and how the blockade of PD-1, which is expressed in T_regs_ [96,97,98], could affect the activity of these cells is more controversial. In fact, while some reports show that the pharmacological blockade of PD-1 in T_regs_ may enhance the activity of these cells, others reported opposite results [99,100]. In conclusion, it is not surprising that ICI hepatotoxicity can be associated with NASH, since they may enhance the immune mechanisms behind this disease. From this perspective it is tempting to speculate that ICIs could cause clinical NASH by altering the equilibrium between immune activation and its suppression in patients already at risk for this disease.

## 7. Implications for Hepatocellular Carcinoma

A special group of patients who are potential candidates for immunotherapy is those with hepatocellular carcinoma (HCC), which usually arises in the context of cirrhosis, but in about 20% of cases it can occur in its absence [101]. Pembrolizumab and nivolumab (as single agents or in association with ipilimumab) have been approved for the treatment of HCC by the FDA—but not by the EMA—based on the positive results of preclinical and clinical studies [102,103,104,105]. In HCC patients, the issue of ICI-induced hepatoxicity appears especially relevant because of its potential consequences on the function of the liver, which is already impaired by the underlying disease or by therapeutical interventions that have been performed to treat the tumor. It is important, however, to underline that available evidence suggests that HCC patients are not more susceptible to developing ICI-induced hepatotoxicity than those affected with other types of cancer [106].

HCC is a heterogenous disease which can be caused by multiple factors, including hepatitis virus B or C infection; alcohol consumption; toxin exposure; and most importantly, NAFLD [107]. Even though the exact prevalence of HCC in the setting of NAFLD is uncertain, it is estimated to range from 2.4% to 38% [108], and a recent cross-sectional study established that patients with NASH have a 60% higher probability of developing HCC than the general population [109]. It is worth emphasizing here that NAFLD is the most rapidly raising causative factor for liver transplantation in patients with HCC in the US, and therefore, its contribution to HCC occurrence is expected to become more and more relevant in the years to come [110]. Mechanistically, the association between NAFLD and HCC is explained by assuming that NAFLD, by progressing from steatosis to NASH and eventually to cirrhosis, could cause the release of large amounts of proinflammatory cytokines and growth factors to induce a microenvironment favorable for HCC development. It has been demonstrated, for instance, that TNF-α not only increases inflammation but also directly promotes HCC growth by acting on TNF-R1 receptors on cancer cells [111]. Likewise, IL-6 and IL-17 promote hepatocellular carcinoma by hepatocyte apoptosis and elevating cell proliferation [112,113]. In addition, cytokines—particularly IL-17 [114]—and insulin resistance and the concomitant hyperinsulinemia, commonly found in NAFLD, may further contribute to HCC development (insulin may act as a growth factor for liver cancer cells) [115]. The functional impairment of hepatocytes could decrease their detoxicating activity and enhance the concentrations of cancerogenic substances and free oxygen radicals [116]. Not only might these toxic substances promote DNA damage and cancerogenesis, but they are also responsible for the loss of intrahepatic CD4+ (but not of CD8+) T-lymphocytes, which could contribute to the genesis of HCC in patients affected with NAFLD, by locally impairing immune surveillance against cancer [117]. Altogether, these observations suggest that the immunological mechanisms involved in NASH pathogenesis also have a part in the genesis of NAFLD-related HCC and might confer specific biological properties to this subtype of HCC making it different from the others. By using preclinical models of NAFLD-induced HCC, Pfister et al. (2021) [118] recently investigated whether the peculiar immunological properties of this form of liver cancer could make it differently responsive to ICIs. The main finding of their relevant study was that in NAFLD-induced HCC, liver infiltrating lymphocytes are often exhausted, and therefore less prone than normal to respond to ICIs. Nonetheless, these lymphocytes are still able to maintain the tissue inflammation responsible for HCC development. The pathogenetic role in HCC of the exhausted lymphocytes found in NASH was confirmed by the paradoxical evidence that, in mice with diet-induced NAFLD, a preventive treatment with anti-PD1 antibodies aggravated tissue damage and increased the occurrence of liver tumors, whereas the depletion of CD8+ T-cells protected the mice from HCC. These findings could be translated to humans HCC, as suggested by the evidence that lymphocytes from patients with NAFLD-induced HCC have gene signatures similar to those observed in mice. Moreover, a metanalysis of three phase III trials on ICIs in advanced HCC (CheckMate-45911, IMbrave1505 and KEYNOTE-24010) showed no benefit of ICIs in the subgroup with NAFLD-related HCC and an improved survival in those with HCV- or HBV-related HCC. Eso et al. [119] recently published a metanalysis on the differences in ICIs response in viral and in non-viral HCC, and they included studies that were not examined by Pfister et al. (CheckMate 040, Study 22, GO30140). They concluded that new evidence is needed because the available data do not clearly distinguish among the different forms of non-viral HCC, and therefore, the observed effects could be non-specific for NASH. If further studies confirm the current evidence, NAFLD could represent a factor which contraindicates the use of immunotherapy, an additional piece in the puzzle of the intricate connection between steatosis, steatohepatitis and ICIs we went through in the present review. Efforts are needed to improve the efficacy of ICIs in this group of HCC patients. The combination of ICIs with anti-VEGF drugs, which is nowadays suggested as first-line treatment in HCC patients by ESMO [120], could help achieve this target. Indeed, it has been shown that, upon VEGF blockade, the intratumoral infiltration of cytotoxic T lymphocytes is enhanced, whereas that of regulatory T-cells is decreased [121]. We did not find, however, any published study specifically addressing NASH-related HCC.

## 8. Conclusions

**Figure 1:**
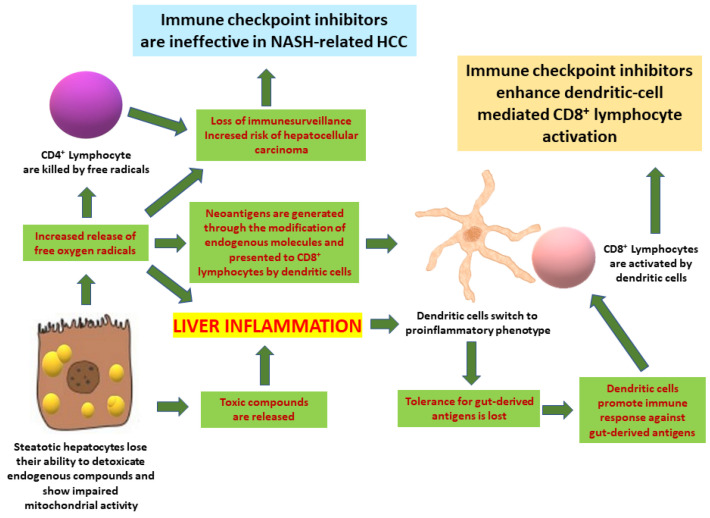
Interrelationship between steatohepatitis and pharmacological effects of immune checkpoint inhibitors in the liver.

By reviewing the literature about the relationship between NAFLD, in its various forms, and cancer immunotherapy, we showed an intricate connection between the two (Figure 1). Available data show that not only may steatosis and steatohepatitis be among the histopathological manifestations of ICI-induced liver toxicity, but NAFLD might also be a risk factor for the hepatotoxicity induced by these drugs. In addition, in the presence of NAFLD, the clinical responses to ICIs could be impaired in patients with HCC. Circumstantial evidence suggests that the lowest common denominator among these different implications of NAFLD in ICI pharmacology could be the presence of shared mechanisms in the pharmacological actions of ICIs and the pathogenesis of NASH.

## Figures and Tables

**Table 1:** Hepatotoxicity of the approved immune checkpoint inhibitors.

	Mechanism of Action	Approved Clinical Indications	Clinical Presentation (Incidence in Clinical Trials/Time to Onset)	References
**Ipilimumab**	Anti-CTA-4 IgG1 human mAb	Melanoma, Renal Cell Carcinoma, CRC, HCC, NSCLC	-Transaminase elevation (34%/3–9 weeks) -Acute hepatitis (1–2%/3.8 months) -Steatohepatitis (NA) -Cholestatic hepatitis (NA)	[14,15,16,17,18,19,20,21,22,23,24]
**Celiplimab**	Anti-PD1, IgG4 human mAb	Cutaneous Squamous Cell Carcinoma, Basal Cell Carcinoma, NSCLC	-Acute hepatitis (2%)	[25,26,27]
**Pembrolizumab**	Anti-PD1, IgG4 humanized mAb	Melanoma, NSCLC, SCLC, HNSCC, Classical Hodgkin Lymphoma, Primary Mediastinal Large B-Cell Lymphoma, Urothelial Carcinoma, Microsatellite Instability-High or Mismatch Repair Deficient Cancer, Microsatellite Instability-High or Mismatch Repair Deficient CRC, Gastric Cancer, Esophageal Cancer, Cervical Cancer, HCC, Renal Cell Carcinoma, Tumor Mutational Burden-High, Cancer Cutaneous Squamous Cell Carcinoma, Triple-Negative Breast Cancer	-Transaminase elevation (27%) -Acute hepatitis (0.7%/3.8 months) -Steatohepatitis (NA) -Cholestatic hepatitis (NA) -Sclerosing cholangitis (NA)	[21,28,29,30,31,32,33,34,35]
**Nivolumab**	Anti-PD1, IgG4 human mAb	Melanoma, NSCLC, Malignant Pleural Mesothelioma, Classical Hodgkin Lymphoma, Urothelial Carcinoma, CRC, Esophageal Squamous Cell Carcinoma	-Transaminase elevation (monotherapy: 7.3%/2.3 months; in combination with ipilimumab: 29.5%/1.5 months) -Acute Hepatitis (monotherapy: 1.8%/3.3 months; in combination with ipilimumab: 7–13%-2.1 months) -Steatohepatitis (NA) -Cholestatic hepatitis (NA) -Sclerosing cholangitis (NA)	[20,21,35,36,37,38,39,40,41,42,43]
**Atezolizumab**	Anti PDL-1 IgG1 human mAb	Urothelial Carcinoma, NSCLC, Triple-Negative Breast Cancer, SCLC, HCC, Melanoma	-Transaminase elevation (common) -Acute hepatitis (1.8%/1.5 months) -Rapid progression of liver fibrosis (NA)	[44,45,46,47]
**Durvalumab**	Anti PDL-1 IgG1 human mAb	NSCLC, SCLC	-Transaminase elevation (8.1%) -Acute hepatitis (0.8%)	[35,48,49,50,51,52,53]
**Avelumab**	Anti PDL-1 IgG1 human mAb	Merkel carcinoma, Urothelial carcinoma, Renal carcinoma	-Transaminase elevation (common) Acute hepatitis (monotherapy: 0.9%/2.5 months; in combination with axitinib: 7%/2.8 months)	[35,54,55,56,57]

Abbreviations: mAb: monoclonal antibody; NSCLC: non-small cell lung cancer; SCLC: small cell lung cancer; HNSCC: head and neck squamous cell carcinoma; HCC: hepatocellular carcinoma; NA: not available.

## Data Availability

Not applicable.
